# Long non-coding RNA H19 alleviates hippocampal damage in convulsive status epilepticus rats through the nuclear factor-kappaB signaling pathway

**DOI:** 10.1080/21655979.2022.2074760

**Published:** 2022-05-21

**Authors:** Yangmei Xie, Ming Wang, Xiaolin Deng, Yinghui Chen

**Affiliations:** a Department of Neurology, Huashan Hospital, Fudan University, Shanghai, China; b Department of Neurology, Xiehe Hospital, Tongji Medical College, Huazhong University of Science and Technology, Wuhan, China

**Keywords:** Inflammation, microglia, convulsive status epilepticus, H19, NF-κΒ signaling pathway, hippocampal damage

## Abstract

Previous studies have demonstrated that inflammation plays a critical role in hippocampcal damage and cognitive dysfunction induced by convulsive status epilepticus (CSE). Emerging evidence indicated that the long non-coding RNA (lncRNA) H19 acts as an important regulator of inflammation in various diseases. However, the role of H19 in CSE is still unkonwn. In the present study, pilocarpine-induced SE rat model was used to explore the role of H19 in hippocampal neuron damage in CSE. Our results indicated that the increased level of H19 is positively correlated with the expression of inflammatory cytokines (TNF-α and IL-1β) in hippocampus of SE rats. Moreover, knockdown of H19 could inhibit the activation of microglia and suppress the expression of inflammatory cytokines via nuclear factor-kappaB (NF-κB) signaling pathway. It was further revealed that downregulation of H19 could alleviate hippocampal neuron damage induced by CSE. These findings indicated that H19 modulates inflammatory response and hippocampal damage through the NF-κB signaling pathway in the CSE rats, which provides a promising target to alleviate hippocampcal damage of CSE.

## Graphical Abstract

**Figure:**
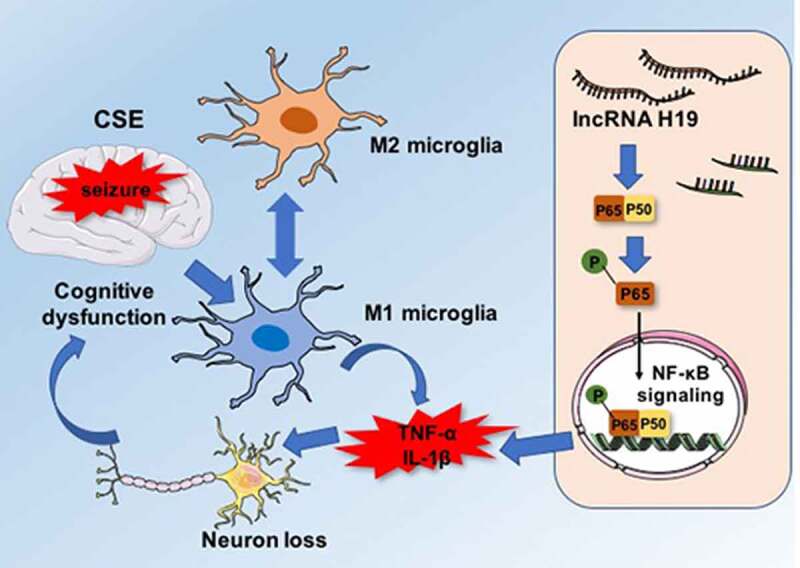

## Highlights

- Upregulation of lncRNA H19 is closely correlated with increased expression of inflammation in hippocampus of CSE rats.
- Konckdown of H19 inhibits the activation of microglia and expression of inflammation.
- Konckdown of H19 alleviates neuronal loss and cellular apoptosis through NF-κB signaling pathway in hippocampus of CSE rats.

## 1. Introduction

Convulsive status epilepticus (CSE) is one of the most challenging and life-threatening neurologic emergencies, which is induced by abnormal termination or initiation dysfunction of epileptic seizures [1]. In the clinic, combination of antiseizure medictions (ASMs) might abolish acute seizures in most cases of CSE. Nevertheless, it can hardly prevent the long-term complications in survivors of CSE such as hippocampal damage and cognitive dysfunction [2].

The mechanism underlying hippocampal damage in CSE is complicated. Previous findings demonstrated that neuroinflammation is one of the most important factors in the initation and development of CSE [3]. Excessive proinflammatory cytokeines can alter blood-brain barrier integrity and disturb neurotransmitter homeostasis, which may cause poor efficacy of ASMs [4]. Additionally, the outbreak of inflammatory response may induce neuronal hyperexcitability and sustained seizures, which may result in neuronal degeneration and long-term cognitive dysfunction [5]. Previous data demonstrated that anti-inflammatory targets or treatments could reduce seizure frequencies and alleviate brain injury of CSE [6,7]. Nuclear factor-kappaB (NF-κΒ) transcription factor is one of the key regulators of inflammatory and immune responses [8]. Additionally, NF-κB sinaling pathway is the crosstalk of various signaling pathways, like TGF-β, mTOR and MAPK signaling pathways, which could rapidly cause inflammatory cascade reaction [9]. It was found that the increased activation of NF-κB contributes to dysfunction of neuronal circuits and neuron loss in the hippocampus of CSE rats [10]. However, exogenous drugs targeting inflammatory mechanisms have largely failed to be translated into the clinic due to lack of specificity and side effects. Therefore, identifying endogenous molecules that modulate inflammation may provide an alternative strategy.

Recently, emeging evidence indicated that the long non-coding RNAs (lncRNAs) emerge as promising regulators of inflammatory reponse in various diseases [11], which can regulate gene expression via diverse mechanisms, including gene imprinting, dosage compensation, transcriptional or posttranscriptional processing. Additionally, it was found that lncRNAs are involved in the regulation of pathological processes of epilepsy [12]. Emerging studies have revealed that the lncRNA H19 plays a critical role in the pathogenesis, treatment, and even prognosis of neurological disorders [13]. The lncRNA H19 is an imprinted gene, located on human chromosome 11 and is transcribed from the maternally inherited allele, which is evolutionary conserved in mammals [14]. It was imiplied that H19 could intiate inflammation and neuronal death in retinal ischemia damage [15]. Additionally, previous study reported that lncRNA H19 was overexpressed in the rat model of temporal lobe epilepsy, and its downstream targets were predicted to be related to immune and inflammatory responses and neuronal apotosis by function and pathway analysis [16]. Consistent with previous study, our study found that H19 was significantly increased in the hippocampus of CSE rats by high-throughput sequencing (Table S1). However, the mechanism of H19 in regulation of inflammation and neuron injury in CSE is stilled to be eculicated. We therefore speculated that H19 is involved in hippocampal damage induced by increased inflammation in CSE. The present study was aimed to explore the role of H19 in the regulation of inflammation and hippocampal damage in the model of CSE.

## 2. Materials and methods

### 2.1 Establishment of convulsive status epilepticus (CSE) rat models

Male Sprague-Dawley (SD) rats weighing 160–180 g were purchased from the Animal Center of Fudan University (Shanghai, China). All animal experiments were approved by the Ethics Committee for Animal Care of Huashan Hospital of Fudan University. Lithium-pilocarpine induced CSE models were established by intraperitoneal injection of pilocarpine as described previously [17]. CSE models were regarded as successfully kindled when the rats developed seizure scoring grade IV–V and exhibited a sustained state. SE in rats was continuously monitored and terminated after 60 min by intraperitoneal of diazepam.

### 2.2 Grouping and stereotaxic injection of the AAV vectors

H19 was knocked down using an adeno-associated virus (AAV) delivery system according to previous paper [18]. The AAV9 vectors carrying short hairpin RNA targeting H19 (sh-H19) and the empty AAV9 vectors (sh-Vec) were constructed by Obio Technology Co., Ltd. (Shanghai, China). AAV9 vectors were sereotaxically injected into both the dorsal and ventral hippocampus 21d before SE induction. A total of 6 μl AAV9 vectors was infused through a microsyringe at a speed of 0.2 μl/min into the dorsal hippocampus (3.3 mm posterior to bregma, 2.2 mm lateral from midline, 3.0 mm below to dura) and the ventral hippocampus (5.2 mm posterior to bregma, 4.8 mm lateral from midline, 6.0 mm below to dura, 3 μl at each location). To prevent backflow of viral particles, the pipette was left in place for an additional 5 min after injection. The rats of normal group and CSE group were all received an equal amount of saline as the sham-operated controls.

### 2.3 Western blot analysis

Total protein was extracted from rat hippocampal tissues with SDS lysis buffer (Beyotime, Shanghai, China). The proteins (15 μg) were separated by SDS-PAGE electrophoresis and transferred the PVDF membrane. The PVDF membrane was blocked with 5% nonfat milk for 1 h at room temperature. Then the membrane was washed thrice with TBST for 15 min each time and incubated with the primary antibody for p-p65, p65 IL-1β or TNF-α overnight at 4°C, followed by washes with TBST and incubation with HRP-conjugated Affineur goat anti-rabbit IgG (H + L) for 1 h at room temperature. The anti-GAPDH (Proteintech Group, United States) was used as an internal quantitative control. Reactive bands were detected using ECL-Plus reagent (Merck Millipore, Darmstadt, Germany), and the band intensity was quantified using a Bio-Rad 2000 gel imaging system equipped with QUANTITY ONE software (Bio-Rad Laboratories, Hercules, CA, United States).

### 2.4 Quantitative Real-Time PCR Analysis (qRT-PCR)

Total RNA was extracted from rat hippocampal tissues using TRIZOL Reagent (Takara, Japan). The cDNA of corresponding genes were synthesized using Prime Script reagent kit and was measured using SYBR Premix Ex Taq (Tli RNaseH Plus; Takara), with GAPDH as an internal control in an Applied Biosystems 7300 instrument (Applied Biosystems, Foster City, CA, United States). The primers used in the study are listed in Table 1.

**Table 1.** ~TC~

Gene	Primer	Sequences (5´ to 3´)
TNF-α	Forward	AAAGGACACCATGAGCACGGAAAG
	Reverse	CGCCACGAGCAGGAATGAGAAG
IL-1β	Forward	GCCAACAAGTGGTATTCTCCA
	Reverse	TGCCGTCTTTCATCACACAG
GAPDH LncRNA H19	Forward Reverse Forward Reverse	GATTTGGCCGTATCGGAC GAAGACGCCAGTAGACTC GATGGAGAGGACAGAAGGACAGT GAGAGCAGCAGAGATGTGTTAGC

### 2.5 Immunofluorescence analysis

The brain slices were dried, dewaxed and permeabilized with 0.1% Triton X-100. Then brain slices were blocked in 5% goat serum, and incubated with anti-Iba primary antibody (1:200 dilution; Cell Signaling Technology, United States) overnight at 4°C. Then the slices were washed and incubated with goat secondary antibodies conjugated with Alexa Fluor 594 (Life Technologies, USA) and treated with the antifade mountant with DAPI (Shanghai Weiao Biological Technology Co., Ltd, China). The images were captured using a confocal laser scanning microscope (Leica, Germany).

### 2.6 HE staining

The brain tissues of rats were fixed with 4% paraformaldehyde (Shenggong, Shanghai, China), washed, dehydrated, transparentized, immersed in wax, and cut into slices. The dried sections were immersed in a dyeing vessel containing xylene I and xylene II for dewaxing. Sections were successively immersed in different concentrations of alcohol, double-distilled water to hydrate, stained in HE, dehydrated, and sealed. The prepared tissue sections were observed under an optical microscope and photographed using a microimaging system.

### 2.7 Nissl staining

Brain sections were stained with 1% toluidine blue (Wokai Co. Ltd, China) for 10 min. The slides were then rinsed in distilled water, dehydrated in a series of gradient ethanol, cleared in xylene, and cover slipped with neutral balsam. The total cell number in CA1 and CA3 areas of the hippocampus were counted from three non-overlapping fields of each section (Nikon Eclipse 50i, Japan) using a computer-assisted image analysis system (NIS-Element Analysis software). The cells in well delimited form with a distinct nucleus were counted. Neurons with shrunken cell body or surrounding empty spaces were considered destined to die and excluded from the counting.

### 2.8 Statistical analysis

All data were analyzed using SPSS 17.0 (SSPS, Inc., Chicago, IL, USA) . The results are expressed as mean ± SD. Comparisons between groups were performed using one-way analysis of variance and *t*-test. Pearson correlation analysis was used for two-variable correlation analysis. *P* < 0.05 was considered to be statistically significant. All experiments were repeated at least three times.

## 3. Results

### 3.1 Expression of inflammatory cytokines was increased in the hippocampus of CSE rats

**Figure 1.**
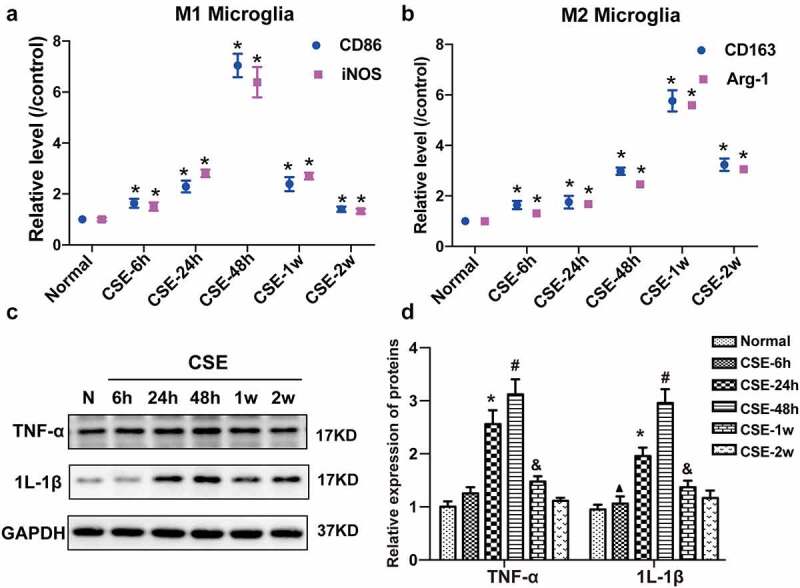
**The expression of inflammatory cytokines in the hippocampus of CSE rats**. (a) The expression of M1 microglia markers at different time point after CSE induction. (b) The expression of M2 microglia markers at different time point after CSE induction. (c) The representative protein bands of TNF-α and IL-1β in the hippocampus of CSE rats at different time point after CSE induction. (d) Quantification of protein levels for TNF-α and IL-1β. (▲P < 0.05 vs CSE-6 h; *P < 0.05 vs CSE-24 h; #P < 0.05 vs CSE-48 h; &P < 0.05 vs CSE-1 w).

Microglia are the main immunomodulatory cells in the central nervous system. A large number of studies have indicated that thedysregulated polarization of microglia contributes to the development CSE. Microglia are mainly divided into two phenotypes including M1 or M2 polarized microglia according to the different function [19]. M1 polarized microglia are demonstrated to release various proinflammatory cytokines and induce neuron injury. Whereas M2-activated microglia release numerous anti-inflammatory cytokines or neurotrohhic factors in the brain, which play a neuronprotective role through promoting phagocytosis and regenration [20]. To investigate the change of microglia polarization in the brain of CSE, the markers of M1 microglia and M2 microglia were detected by PCR at different stage of CSE. It was observed that the M1 microglia markers significantly increased in acute stage of CSE (6–48 h), but decreased gradually to baseline 2 week after SE induction (Figure 1(a)). Whereas, the M2 microglia markers were not significantly increased in acute stage of CSE (6–48 h), but increased obviously at sub-acute stage of CSE (1–2 w) (Figure 1(b)). The expression of inflammatory cytokines (TNF-α and IL-1β) in the hippocampus of CSE rats was detected at different time after CSE induction (6 h, 24 h, 48 h, 1 w and 2 w). The results showed that the level of TNF-α and IL-1β in the hippocampus of CSE rats was increased at 24 h, up to the maximum at 48 h, but decreased gradually to baseline at 2 week after CSE induction (Figure 1(c,d)). The result indicated that the M1microglia were rapidly activated and cause an intense inflammatory cascade at acte stage of CSE.

### 3.2 The level of H19 was positively correlated with inflammatory cytokines in the hippocampus of CSE rats

**Figure 2.**
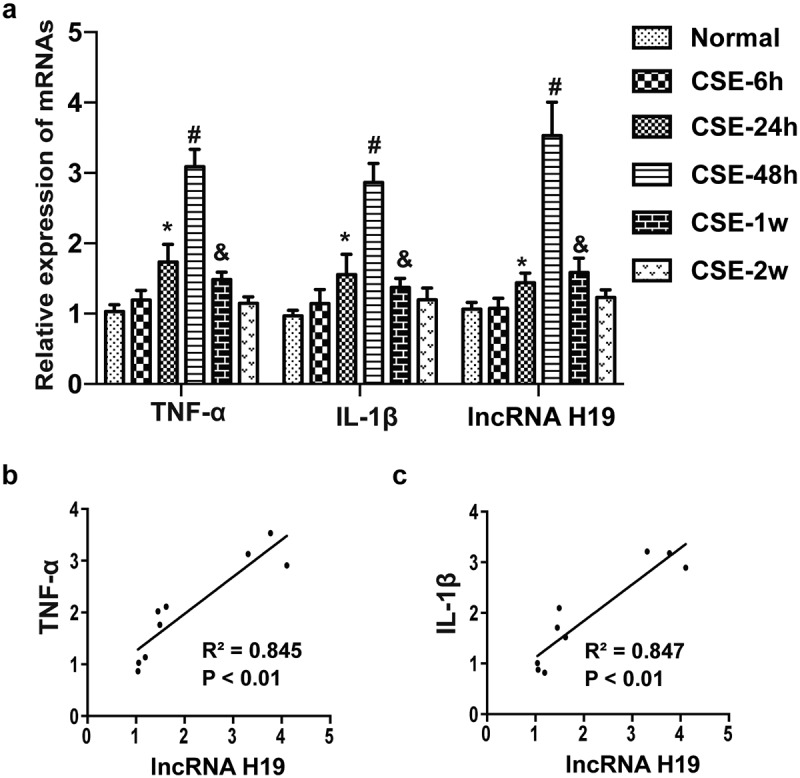
**The expression of H19 was positively correlated with inflammatory cytokines**. (a) The expression of H19 and inflammatory cytokines (TNF-α and IL-1β) in the hippocampus of CSE rats at different time point after CSE induction. (b) Pearson correlation analysis between H19 and TNF-α (R2 = 0.845, p < 0.01). (c) Pearson correlation analysis between H19 and IL-1β (R2 = 0.847, p < 0.01). (*P < 0.05 vs CSE-24 h; #P < 0.05 vs CSE-48 h; &P < 0.05 vs CSE-1 w).

Previous studies have indicated that lncRNA H19 was related to immune and inflammatory response in epilepsy. To explore the correlation of H19 and inflammation in the hippocampus of CSE rats. The gene expression of inflammatory cytokines (TNF-α and IL-1β) and H19 in the hippocampus of CSE rats was detedcted by qRT-PCR. The results showed that the expression of TNF-α and IL-1β was increased at 24 h, up to the maximum at 48 h, but decreased gradually to baseline at 2 week after CSE induction. Interestingly, the change of H19 level showed a similar trend with the inflammatory cytokines(Figure 2(a)). Pearson correlation analysis showed that the level of H19 was positively correlated with TNF-α and IL-1β (Figure 2(b,c) R2 = 0.845, p < 0.01; R2 = 0.847, p < 0.01; respectively). The finding indicated that H19 was closely associated with abnormal expression of inflammatory cytokines in the hippocampus of CSE rats.

### 3.3 Knockdown of H19 inhibited microglia activation in the hippocampus of CSE rats

**Figure 3.**
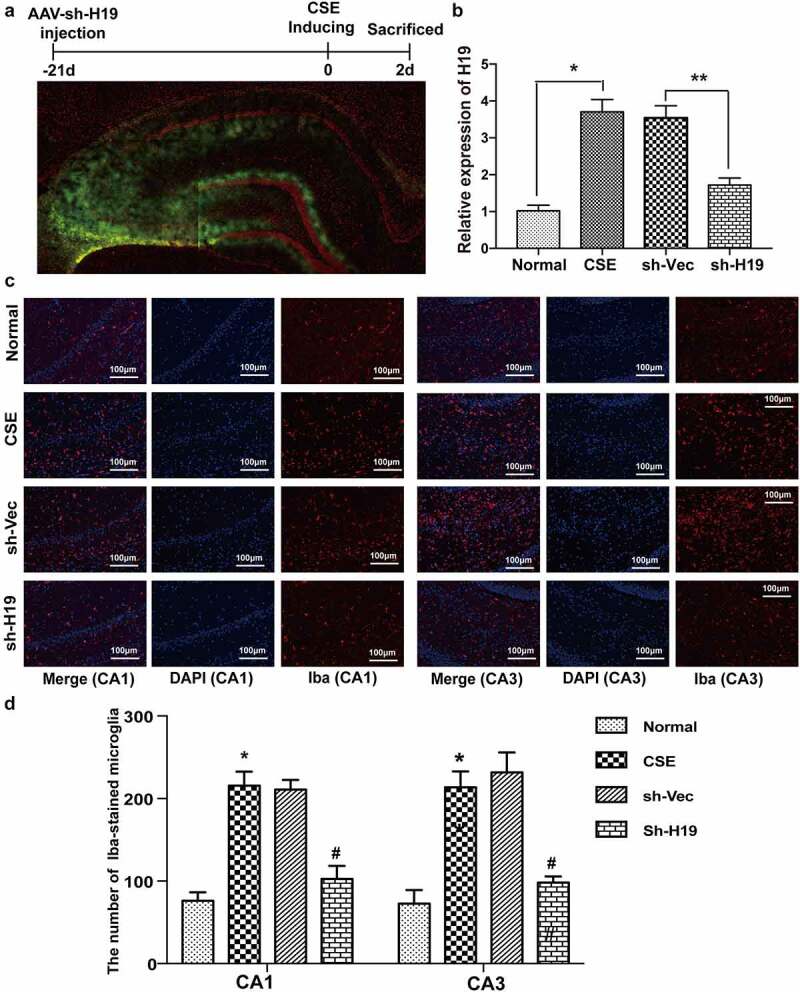
**Knockdown of H19 inhibited microglia activation in the hippocampus of CSE rats** (a) Representative distribution images of sh-H19 vectors in the hippocampus of CSE rats by fluorescence microscope. (b) The knockdown efficiency of sh-H19 detected by qRT-PCR. (c) Representative images of Iba fluorescence around microglia in the hippocampus of CSE rats. (d) The counts of Iba-stained microglia in CA1 and CA3 area of hippocampus in CSE rats. (*P < 0.05 CSE vs Normal, #*P* < 0.05 sh-H19 vs sh-Vec).

A large number of studies have confirmed that microglia mediated inflammation plays a key role in the brain of CSE. To investigate the role of H19 on microglia activation, H19 was knocked down using an adeno-associated virus (AAV) delivery system. The results of fluorescence observation on brain slices showed that the injected vectors had fused into the hippocampus (Figure 3(a)) 21d after injection. The results of qRT-PCR showed that the injection of sh-H19 could reduce H19 expression over 50% in the hippocampus of CSE rats (Figure 3(b)). Iba is one of the specific molecular markers around microglia surface which represents microglia activation. The result of immunofluorescence staining showed that Iba fluorescence was increased obviously in CA1 and CA3 area in the hippocampus of CSE rats, while knockdown of H19 reduced Iba fluorescence in CA1 and CA3 area of hippocampus in CSE rats (Figure 3(c,d)). The results indicated that knockdown of H19 could inhibit microglia activation in the hippocampus induced by CSE.

### 3.4 Knockdown of H19 inhibited the expression of inflammatory cytokines via NF-κB signaling pathway in the hippocampus of CSE rats

**Figure 4.**
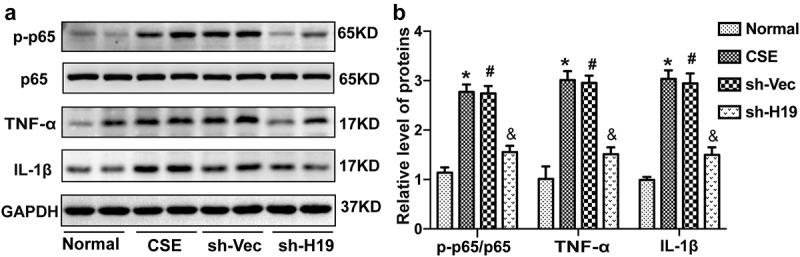
**Knockdown of H19 inhibited the expression of inflammatory cytokines via NF-κB signaling pathway in the hippocampus of CSE rats**. (a) The representative protein bands of TNF-α, IL-1β, p-p65 and p65 detected by Western blot analysis in the hippocampus of CSE rats. (b) Quantification of protein levels for TNF-α, IL-1β, p-p65/p65 in the hippocampus of CSE rats. (*P < 0.05 CSE vs Normal, #P < 0.05 sh-Vec vs CSE, &P < 0.05 sh-H19 vs sh-Vec).

NF-κΒ is one of the key transicription factors to induce the expression of classical inflammatory cytokines like TNF-α, IL-1β and so on, which was demonstrated to promote apoptotic neuronal cell death in the hippocampus of CSE [8]. To further explore the role of H19 on the regulation of inflammation and NF-κΒ signaling pathway. The level of inflammatory cytokines and activation of NF-κB (represented by the ratio of p-p65/p65) were examined by Western blot analysis. As shown in Figure 4(a,b), it was observed that the expression of inflammatory cytokines (TNF-α and IL-1β) and activation of NF-κB were increased in the hippocampus of CSE rats. While knockdown of H19 inhibited expression of TNF-α and IL-1β as well as the activation of NF-κB (p-p65/p65) in the hippocampus of CSE rats. The results indicated that knockdown of H19 might inhibit expression of inflammation via NF-κB signaling pathway.

### 3.5 Knockdown of H19 alleviated neuronal loss in the hippocampus of CSE rats

**Figure 5.**
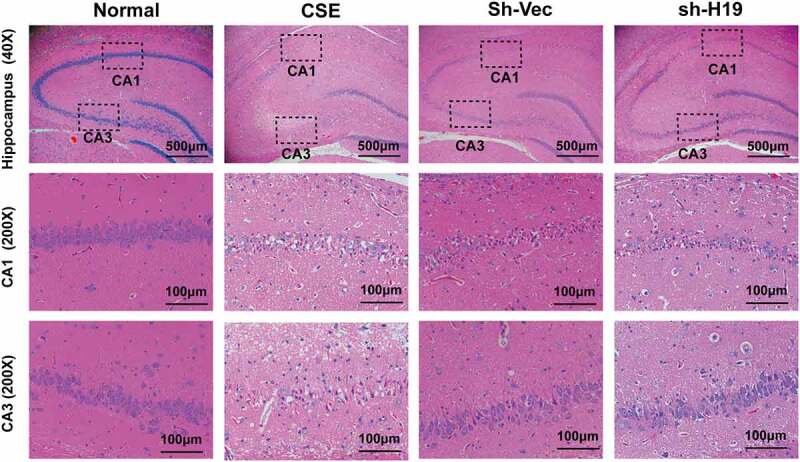
The effect of H19 on morphological changes in the hippocampus of CSE rats.

**Figure 6.**
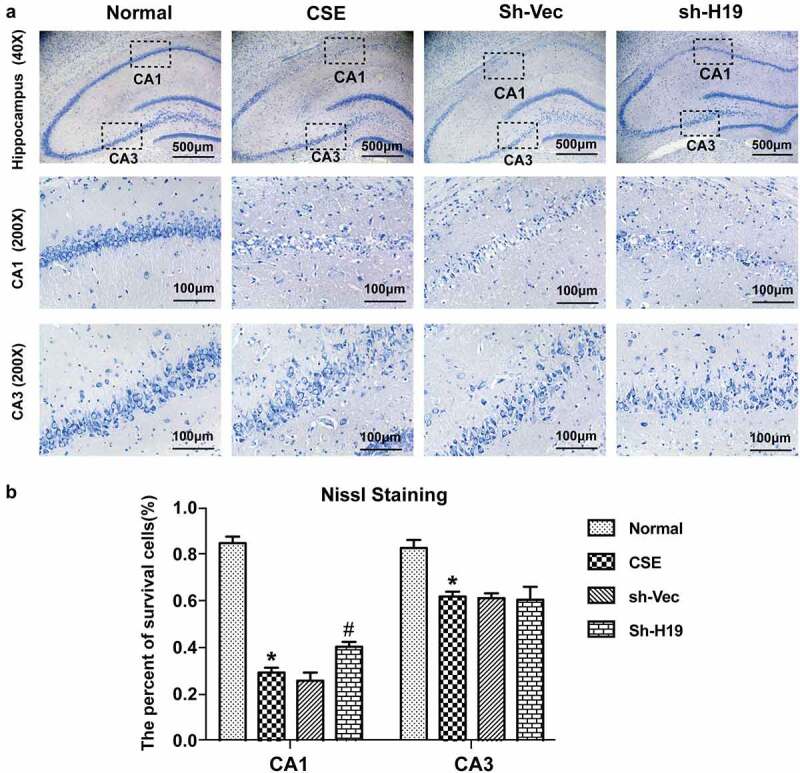
**The effect of H19 on neuronal loss in the hippocampus of CSE rats**. (a) Representative images of Nissl staining in CA1 and CA3 areas in the hippocampus of CSE rats. (b) Quantification of survival neurons in CA1 and CA3 areas in th hippocampus of CSE rats. (*P < 0.05 CSE vs Normal, #P < 0.05, sh-H19 vs sh-Vec).

Neuron damage and cognitive dysfunction have become the great challenge for CSE therapy. To explore the role of H19 on neuron injury and hippocampal damage induced by CSE, HE staining and Nissl staining were conducted. The HE staining was performed to observe the morphological change in the hippocampus of CSE. According to the results of HE staining, neurons in the hippocampus of the CSE group were swollen and loosely arranged compared to the normal group. Additonally, morphological dysfunction including chromatic agglutination, karyopyknosis, and nuclear fragmentation, was obviously observed in the CA1 and CA3 region of hippocampus in the CSE group. Whereas, knockdown of H19 showed a trend of reduction in the morphological dysfunction compared to the sh-Vec group (Figure (5)). The results of Nissl staining displayed that neuron loss was significantly increased in CA1 and CA3 areas in the hippocampus of CSE rats. While knockdown of H19 alleviated neuron loss in CA1 areas of hippocampus in CSE rats, but had no obviously protective effect for neuron damage in CA3 areas (Figure (6)).

## 4. Discussion

Despite the recent advent of additional ASMs and respective surgery, the treatment of CSE remains a major challenge in the clinic. Actucally, medically intractable CSE often carries poor prognosis with a mortality of approximately 20–30% in older adults, and survivors often suffer neurological and cognitive deficits [21,22].

The mechanism underlying CSE-induced chronic brain injury is complicated. Recently, neuroinflammation is recognized a critical factor that contributes to hippocampal damage of CSE [23]. The hippocampus is one of the most susceptible regions to be damaged by seziure-induced ischemia and hypoxia in the brain. The increased level of inflammation induced by sustaining seziures may cause persistent pathological alterations in the hippocampus like neuron degeneration, glial cell proliferation, moss fibrosis budding and abnormal neuronal networks [24], which could eventually induce decliened cognition of patients with CSE. Microglia are the resident immunomodulatory cells in the central nervous system, which paly a key role in the development of CSE [25]. Previous study indicated that M1 polarized microglia in the brain are obviously increased in acute stage of epilepsy, which contributes to continuous seziures and severe neuron loss [26]. Consistnet with previous study, our data found that the M1 microglia markers weresignificantly increased in acute stage of CSE (6–48 h), but the M2 microglia markers were increased obviously at sub-acute stage of CSE (1–2 w) after CSE induction. The results indicated that the M1 microglia were rapidly activated to release a large amount of proinflammatory factors in acute stage of CSE, which results in neuronal hyperexcitability and injury. Therefore, early intervention targeting inflammation in acute stage of CSE is urgently needed to interrupt the continuous seziures and attune the neuron injury.

Recently, emerging studies have demonstrated that non-coding RNA-baesd therapy may paly a significant role in the regulation of inflammatory pathways involved in epilepsy [27]. The lncRNAs can make a fast response to outside stimuli by acting as a center platform to achieve integration of various signaling pathways [28]. Accumulating evidence has indicated that the lncRNA and its related molecular pathways are closely involved in the pathogenesis of epilepsy. It was reported that lncRNA TUG1 can regulate hippocampal neuron cell activity and apoptosis which may be a biomarker of temporal lobe epilepsy diagnosis in children [29]. Additionally, it was found that lncRNA NEAT1 affects inflammatory response via regulating Notch signaling pathway in epilepsy cell model. However, whether the lncRNA can regulate inflammatory pathways and alleviate neuron injury in vivo is still to be clarified. In the present study, it was found that the increased level of H19 was positively correlated with inflammatory cytokines in the hippocampus of CSE rats. And knockdown of H19 could inhibit the expression of inflammatory cytokines in the hippocampus of CSE rats.

However, the underlying mechanism of H19 regulating inflammation in the hippocampus of CSE is still to be explored. NF-κΒ is one of the key regulators of inflammatory and immune responses,which is involved in various physiological and pathological scenarios [30]. Previous studies found that the activity of NF-κB is significantly increased in the brain of CSE rats, which contributes to neuron loss and brain dysfunction. And the specific inhibitor of the NF-κB could alleviate the level of inflammation and the neuronal loss in CSE rats [31]. However, exogenous drugs targeting inflammatory pathways have largely failed to be translated into the clinic due to lack of specificity and side effects. Emerging evidence indicated that the lncRNA may emerge as a novel regulator of NF-κB signaling pathway, which may provide an alternative strategy for CSE [32]. Previous study has reported that lncRNA NKILA regulates endothelium inflammation by controlling NF-κB signaling pathway [33]. In the present study, it was demonstrated that knockdown of H19 could inhibit the activation of NF-κB signaling pathway and alleviate hippocampal damage and neuron loss induced by CSE. It was consistent with the previous data that H19 contributes to glial cell activation and apoptosis of hippocampal neurons in the brain of temporal lobe epilepsy [34,35].

In summary, our data indicated that knockdown of H19 could inhibit the expression of inflammation and alleviatehippocampal damage via NF-κB signaling pathway, which provides a potential therapeutic target for CSE. However, we have to admit there are still some deficiencies in our study. Firstly, further investigation to explore the effect of H19 on electroencephalography (EEG) and cognitive function is urgently supplemented in the model of CSE rats. Secondly, we explored the function of H19 by loss-function of H19, gain-function of H19 is needed to verify the regulatory function of H19. Additionally, the molecular mechanism underlying H19 regulation of NF-κB signaling pathway is needed to be explored in the future.

## 5. Conclusions

In conclusion, our research showed that konckdown of H19 could inhibit inflammation and alleviate nuuron injury through regulating NF-κΒ signaling pathway in the hippocampus of CSE. This finding contributes to a better understanding of the molecular mechanism of CSE, which provides a fundamental basis for novel treatment of CSE in the future.

## Supplementary Material

Supplemental Material

## Abbreviation

CSE: Convulsive status epilepticus
NF-κΒ: nuclear factor-kappaB
ASMs: antiseizure medictions
(AAV): adeno-associated virus
EEG: Electroencephalography

## Data sharing statement

All datasets generated in this study were included in the manuscript. The original data in this manuscript are available from the corresponding author upon reasonable request.
